# Targeted Apoptosis Induction in Oral Squamous Cell Carcinoma by *Goniothalamus umbrosus* : A Pathway Through Bax, Bcl-2, and Caspase 3

**DOI:** 10.1055/s-0045-1813037

**Published:** 2025-12-18

**Authors:** Nuraini Che Aziz, Basma Ezzat Mustafa Alahmad, Muhanad Ali Kashmoola, Widya Lestari, Nik Mohd Mazuan Nik Mohd Rosdy, Khairani Idah Mokhtar

**Affiliations:** 1 Department of Fundamental Dental Medical Science, Kulliyyah of Dentistry, International Islamic University Malaysia, Kuantan, Pahang, Malaysia; 2 Department of Dentistry, Bilad Alrafidain University College, Diyala, Iraq; 3 Faculty of Dentistry, Universiti Teknologi MARA, Sungai Buloh Campus, Jalan Hospital, Sungai Buloh, Selangor, Malaysia

**Keywords:** *Goniothalamus umbrosus*, oral squamous cell carcinoma, *Bax*, *Bcl-2*, caspase-3

## Abstract

**Abstract:**

**Objective:**

Oral squamous cell carcinoma (OSCC) is one of the most aggressive malignancies of head and neck cancer associated with severe morbidity and high mortality rates. While conventional treatment modalities are effective in eliminating cancer cells, they frequently result in adverse side effects. Research continues to explore plant-based therapeutics with selective cytotoxicity against cancer cells. *Goniothalamus umbrosus* is traditionally used among medicinal folks and has shown potential in its anticancer properties. This study aims to explore the role of *G. umbrosus* hexane extract (GUHE) in inducing apoptosis by elucidating its mechanism of action in OSCC (HSC-3) while comparing its effect on human gingival fibroblast (HGF) cells.

**Materials and Methods:**

The expression of proapoptotic gene (Bax) and antiapoptotic gene (Bcl-2) in HSC-3 and HGF cell lines that had been pretreated with GUHE was evaluated by RT-qPCR. The activity of caspase-3 protein was quantified by ELISA assay.

**Results:**

RT-qPCR analysis revealed that GUHE significantly upregulated the *Bax* expression by 1.69-fold and downregulated *Bcl-2* expression by 42% in the HSC-3 cell line. The activity of caspase-3 protein was significantly increased in HSC-3 treated with GUHE. The expression of *Bax* and *Bcl-2* genes and caspase-3 protein activity was not significantly modulated in GUHE-treated HGF.

**Conclusion:**

GUHE selectively induced apoptosis by activating the mitochondrial apoptosis pathway in the HSC-3 cell line without being detrimental to the HGF cell line. This highlights its promising effect as a targeted therapeutic agent for OSCC therapy.

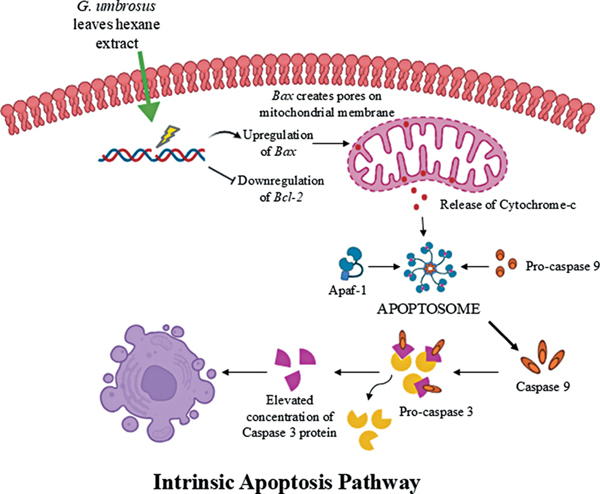

## Introduction

Oral cancer is the 16th most frequently diagnosed cancer worldwide, with 389,846 new cases and 188,438 deaths reported in 2022. 1 The risk factors of oral cancer include alcohol consumption, tobacco use, and betel quid chewing. 2 Oral squamous cell carcinoma (OSCC) accounts for more than 90% of oral cancer and is one of the most aggressive forms of head and neck cancer in the world. 3 Current treatment strategies for patients with OSCC include surgery, chemotherapy, radiotherapy, monoclonal antibody therapy, or a combination of these modalities. 4 Despite advances in the treatment strategy, the prognosis in patients with advanced OSCC remains poor due to the development of various side effects, such as oral mucositis, which further leads to reduced food intake, weight loss, dehydration, inflammation, and treatment interruption. 5 Therefore, it is crucial to investigate potential anticancer agents that can target certain molecular pathways involved in cancer progression and apoptosis.

Apoptosis is a highly regulated form of programmed cell death that plays a fundamental role in maintaining tissue homeostasis by eliminating damaged or unwanted cells. 6 Dysregulation of this pathway is a hallmark of cancer, enabling malignant cells to evade cell death, leading to uncontrolled proliferation. 7 Consequently, therapeutic strategies that restore or enhance apoptotic signaling represent a critical approach in cancer management, offering the potential for selective eradication of tumor cells while minimizing toxicity to normal tissues. Hence, the anticancer agent that can regularly restore apoptosis could be a promising therapeutic agent for cancer therapy.

Research in medicinal plants has garnered increasing attention worldwide for their promising potential in cancer therapy due to their diverse phytochemical with many potential anticancer effects. 8 *Goniothalamus umbrosus* from the Annonaceae family was traditionally used for abortifacient, postpartum healthcare, and as an antipyretic. 9 It has been scientifically reported to exert anticancer effects against breast cancer cells (MCF-7), cervical cancer cells (HeLa), and leukemia cells. 10 11 12 Our previous study demonstrated that *G. umbrosus* induced cell cycle arrest in OSCC cells. 13 However, the effect of *G. umbrosus* and its molecular mechanism of action on OSCC has yet to be explored.

The current study evaluated the anticancer potential of *G. umbrosus* on OSCC, mainly in its ability to modulate the expression of *Bax* and *Bcl-2* as well as activation of caspase-3 protein, thus inducing apoptosis in OSCC cells while comparing its effect on normal healthy HGF cells.

## Materials and Methods

**1. Cell cultures**
The human tongue squamous cell carcinoma (HSC-3) cell line was originally harvested from a metastatic lymph node of tongue squamous cell carcinoma. 14 Human gingival fibroblast (HGF) cell line was primarily excised from the gingiva of the donor (American Type Culture Collection [ATCC]; catalog no.: PCS-201-018). Both cell lines were cultured in DMEM media (ATCC catalog no.: 30-2006) supplemented with 10% FBS (ATCC catalog no.: 30-2021) and 100 U/mL of penicillin/streptomycin (ATCC catalog no.: 30-2300) and maintained at 37°C incubator with a relative humidity of 95 and 5% CO _2_ . The cells were monitored daily under an inverted microscope. The medium was replaced every 48 hours, and the cells were cultured until they reached 80 to 90% confluency.
**2.** ***G. umbrosus*** **extract preparation**
Fresh leaves of *G. umbrosus* were collected from Machang, Kelantan, Malaysia. The plant was verified with voucher number PIIUM 0331, and the herbarium was deposited in the Herbarium, Kulliyyah of Pharmacy, International Islamic University Malaysia, Kuantan, Pahang, Malaysia. The leaves were cleaned, dried at room temperature (25°C) for 3 days, and ground into fine powder. Powdered leaves (100 g) were macerated in 1,500 mL of hexane for 24 hours at room temperature (25°C). 15 The extract was filtered with Whatman No. 1 filter paper, and the process was repeated eight times to confirm thorough extraction. 16 The filtrates were combined, and the solvent was discarded by a rotary evaporator (50°C, 200 mbar).
**3. Gene expression**
Both HSC-3 and HGF cell lines were pre-treated with 176 µg/mL of GUHE (IC _50_ value obtained from the cytotoxicity assay) and incubated for 72 hours. 17 The HSC-3 cells were also treated with 2 µg/mL of cisplatin (Sigma, USA, catalogue no.: P4394) as a positive control. After treatment, total RNA of both cells was extracted using Quick-RNA Miniprep Plus Kit (Zymo Research, United States, catalog no.: R1058) following the manufacturer's instructions. To ensure accurate qPCR results, the concentration (260 nm) and purity (260/280 nm) of RNA samples were quantified using a Nanodrop 2000 spectrophotometer (Thermo Scientific, United States). A ratio of ∼2 was considered pure for the RNA sample. The RT-qPCR analysis was performed by Luna Universal One-Step RT-qPCR Kit (New England Biolabs, United States, catalog no.: E3005S) in a total reaction volume of 20 µL on RotorGene Q (Qiagen, Germany). Glyceraldehyde-3-phosphate dehydrogenase (GAPDH) was used as a housekeeping gene (F: 5′-ACCACAGTCCATGCCATCAC-3′; R: 5′-TCCACCACCCTGTTGCTGTA-3′). The primer sequence of proapoptotic gene, *Bax* , was F: 5′ CAGATGTGGTCTATAATGC 3′; R: 5′ CTAATCAAGTCAAGGTCAC 3′. The primer sequence of antiapoptotic gene, *Bcl-2* , was F: 5′ CCACCAAGAAAGCAGGAAACC 3′; R: 5′ GGCAGGATAGCAGCACAGG 3′. Amplification curves were generated for each primer set prior to the analysis with samples to ensure consistent and reproducible amplification across different concentrations. All experiments were conducted in three independent biological replicates, each using cells from different passages and performed on separate weeks. For qPCR analysis, each biological replicate was further evaluated in three technical replicates to ensure accuracy of the measurement. The non-template control, which was prepared using untreated cells, was also included as a negative control. The comparative ΔΔCt method was used to analyze the expression level of genes.
**4. Preparation of cell lysate**
The HSC-3 and HGF cells were pre-treated with 176 µg/mL of GUHE. 17 Cell lysates were prepared using a mechanical lysis method adopted from a previous study with slight modifications. 18 Briefly, the treated cells were harvested and centrifuged at 1,500 × G for 5 minutes at 4°C. The supernatant was removed, and the pellet was carefully resuspended in ice-cold PBS and centrifuged again at 4°C to remove trypsin residue. After centrifugation, the supernatant was discarded, and 1 mL of ice-cold PBS (0.01 M, pH 7.4) was added to all pellets for both cell lines. The cells were lysed by sonication at 28°C for 30 seconds and repeated four times with 10-second breaks after each cycle. The cells were then incubated at room temperature for 3 hours and occasionally shaken to facilitate the lysing process. The lysed cell suspensions were then centrifuged at 1,500 × G for 10 minutes at 4°C. The supernatant was carefully collected without disturbing the pellet, aliquoted into new microtubes, and quickly stored at −80 °C until ELISA analysis. This was done to avoid multiple freeze–thaw cycles and preserve the protein integrity. Each aliquot was thawed only once and used within 3 days of storage. An amount of 200 µL was retained from the cell suspension and subjected to the Lowry assay using bovine serum albumin as a standard for the estimation of protein concentration. All samples were adjusted to a final concentration of 20 ng/mL before being subjected to a caspase-3 ELISA assay to ensure normalization across replicates.
**5. Caspase-3 activity assay**
The concentration of active caspase-3 in HSC-3 and HGF cell lysates was evaluated using a human CASP3 (caspase-3) ELISA kit (Elabscience Biotechnology Co., Ltd., Hubei, China, catalog no.: E-EL-H6282) according to the manufacturer's protocol. A standard curve was generated using serial dilutions of the caspase-3 standard, ranging from 0.31 to 20 ng/mL, to obtain a range of concentrations. The sensitivity of the caspase-3 assay was 0.19 ng/mL. Absorbance was measured at 450 nm, and the standard curve was plotted by plotting a graph of absorbance against concentration. Sample caspase-3 activity was then determined by interpolation from this standard curve. This assay was conducted in three independent biological replicates ( *n* = 3), each using cells from different passages. Each sample was assessed in duplicate, repeated twice, and the results were averaged for analysis. The optical density was evaluated at 450 nm by using a microplate reader. The expression of caspase-3 in samples was determined by comparing it to the standard curve.
**6. Statistical analysis**
Data were analyzed by IBM SPSS version 29 (Chicago, Illinois, United States) and expressed as mean ± standard deviation (SD). The normality test of the data distribution was assessed using the Shapiro–Wilk test and confirmed to be normally distributed. One-way analysis of variance (ANOVA) with post hoc Bonferroni test was run to analyze the differences between groups. Statistical significance was set at *p* < 0.05.

## Results

**1. Evaluation of apoptosis-related gene expression**

**Fig. 1:**
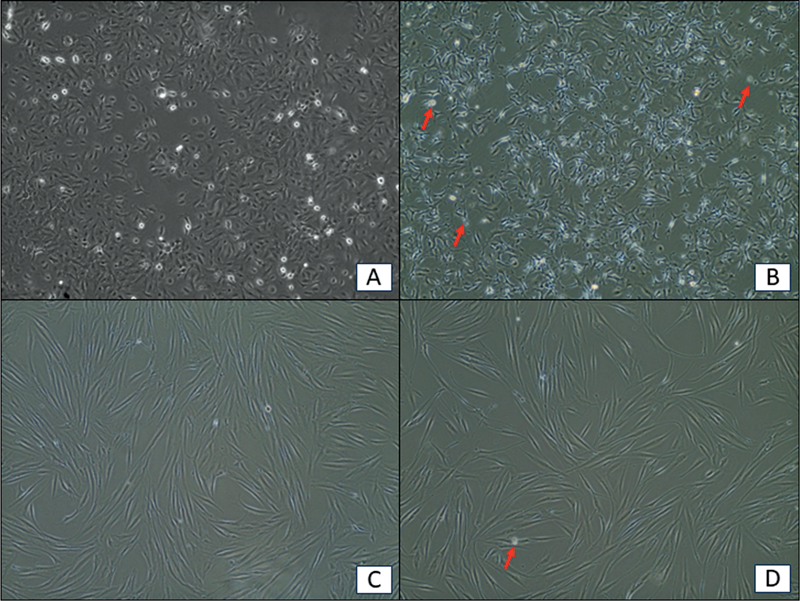
Morphological changes of HSC-3 and HGF cell lines following treatment with GUHE. ( **A** ) Untreated HSC-3 cells show normal morphology. ( **B** ) HSC-3 cells treated with GUHE exhibited marked shrinkage, elongation, and the presence of numerous floating cells, consistent with cell death. ( **C** ) Untreated HGF cells display normal morphology. ( **D** ) HGF cells treated with GUHE show no notable morphological alterations, except for a few floating cells.

Treatment with GUHE has induced cell death in the HSC-3 cell line, with no visual morphological change in the HGF cell line, as observed under an inverted microscope. 17 The morphological changes observed were consistent with cell death, including cell floating, cell shrinking, and cell elongation ( Fig. 1 ). Based on these findings, the current study investigated deeper at the molecular level by conducting gene expression and caspase-3 activity assay to further elucidate the mechanisms underlying the observed cell death.

**Fig. 2:**
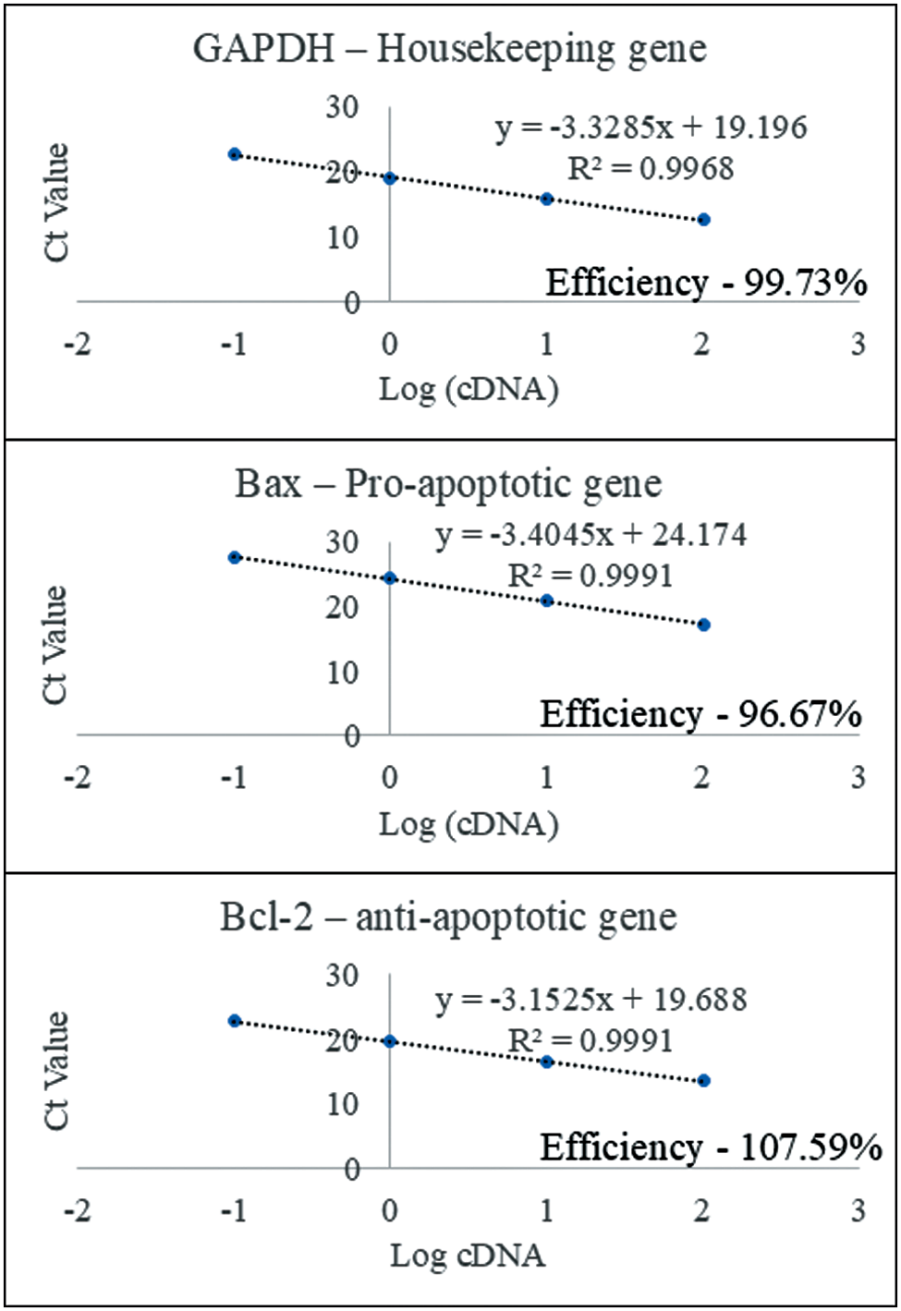
Standard curve generated for GAPDH, *Bax* , and *Bcl-2* primers in qPCR analysis.

Standard curve analysis was performed to validate the efficiency and reliability of the primers used in this study. All primers demonstrated amplification efficiencies within the optimal range of 90 to 110% with GAPDH (99.73%), Bax (96.67%), and Bcl-2 (107.59%) ( Fig. 2 ). The correlation coefficients ( *R* ^2^ > 0.99) indicated excellent linearity of the standard curves, confirming accurate and reproducible quantification.

**Fig. 3:**
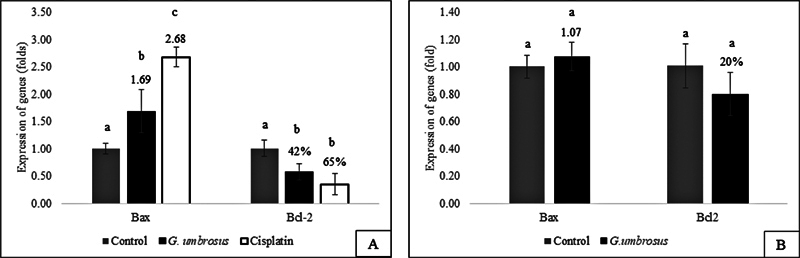
( **A** ) Expression of *Bax* and *Bcl-2* genes in HSC-3 cells treated with GUHE and cisplatin. ( **B** ) Expression of *Bax* and *Bcl-2* genes in HGF cells treated with GUHE. One-way ANOVA with post hoc Bonferroni test. Different alphabet indicates data was significantly different ( *p* < 0.05).

The RT-qPCR analysis revealed significant upregulation ( *p* < 0.05) in the expression of *Bax* in HSC-3 cells treated with GUHE by 1.69-fold compared to untreated cells ( Fig. 3A ). This result was in line with the significant upregulation ( *p* < 0.05) of *Bax* in HSC-3 cells treated with positive control, cisplatin ( Fig. 3A ). Meanwhile, treatment with GUHE also significantly downregulated ( *p* < 0.05) the expression of *Bcl-2* by 42% compared to control ( Fig. 3A ). This finding was also consistent with the significant downregulation ( *p <* 0.05) of *Bcl-2* in HSC-3 cells treated with cisplatin ( Fig. 3A ).

In addition, the expression of *Bax* and *Bcl-2* was also quantified in HGF cells treated with GUHE to evaluate its effect on normal cells. It was revealed that GUHE insignificantly ( *p >* 0.05) upregulated the expression of *Bax* by 1.07-fold and downregulated the expression of *Bcl-2* by 20% in HGF cells ( Fig. 3B ).

**2. Assessment of caspase-3 activity**

**Fig. 4:**
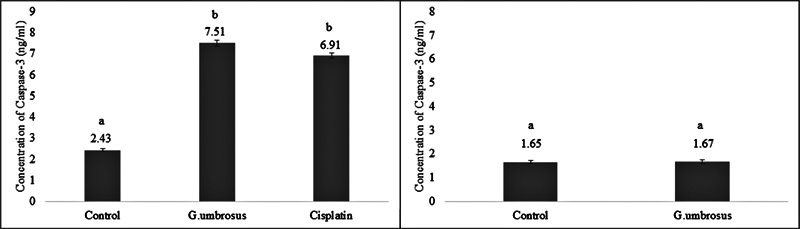
Concentration of caspase-3 in HSC-3 cells significantly increased with GUHE and cisplatin treatment. In HGF cells, GUHE had no significant effect. One-way ANOVA, post hoc Bonferroni test. Different alphabet indicates data was significantly different ( *p* < 0.05).

The activation of caspase-3 was evaluated by ELISA assay following treatment of GUHE in HSC-3 and HGF cell lines for 72 h. It was revealed that caspase-3 protein was significantly expressed ( *p <* 0.05) in HSC-3 cells treated with GUHE and cisplatin with caspase-3 protein concentrations of 7.51 and 6.91 ng/mL, respectively, as compared to control (2.43 ng/mL; Fig. 4 ). The expression of caspase-3 protein in HSC-3 cells treated with GUHE was not significantly different as opposed to HSC-3 cells treated with cisplatin. Meanwhile, the expression of caspase-3 protein in HGF cells treated with GUHE was not significantly different from untreated controls ( *p >* 0.05; Fig. 4 ).

## Discussion

Oral cancer is the most common neoplasm of the head and neck, originating from the tissue of the mouth and throat. It usually affects the squamous cell lining of the oral cavity, with 90% of oral cancer cases being squamous cell carcinoma. 4 Oral cancer therapy, such as surgery, radiotherapy, and chemotherapy, often has limitations and complications, leading patients to seek alternative natural remedies. However, the information on the efficacy of natural remedies is still deficient due to the lack of a scientific approach.

*G. umbrosus* was traditionally used for abortifacient, postpartum healthcare, and antipyretic. 9 Our previous cytotoxicity study revealed that GUHE was moderately selective in inhibiting the proliferation of the HSC-3 cell line, with minimal effect on the HGF cell line, by exerting moderate cytotoxicity against the HSC-3 cell line and low cytotoxicity on the HGF cell line. 17 These findings highlight the potential of GUHE for further analysis as an adjuvant or alternative treatment in oral cancer therapy.

The current study aims to elucidate the apoptotic mechanisms underlying the cytotoxic effects of GUHE by targeting the key apoptotic regulators, *Bax* and *Bcl-2* gene expression, as well as the activation of caspase-3 protein, to provide a more comprehensive understanding of the molecular pathways involved in HSC-3 and normal HGF cells.

Apoptosis is a programmed cell death mechanism that plays a pivotal role in maintaining cellular homeostasis and eradicating malignant cells. 19 This pathway is highly regulated by key proteins such as proapoptotic *Bax* and antiapoptotic *Bcl-2,* and is recommended as a novel approach to developing anti-cancer agents. 20 The balance between these proteins determines the fate of cells, where *Bax* promotes and *Bcl-2* opposes cell death. 21 The enhanced expression of *Bax* increases the permeability of the mitochondrial membrane, leading to the release of cytochrome c from mitochondria to cytosol and further activating the caspases, which are the executioners of apoptosis. 21 22 Meanwhile, the *Bcl-2* protein is often overexpressed in cancer cells, allowing it to escape apoptosis pathways and keep on proliferating. 23

It was found that GUHE induced apoptosis by significantly upregulating the expression of pro-apoptotic gene, *Bax* , and downregulating the expression of antiapoptotic gene, *Bcl-2* . These findings suggest that GUHE induced apoptosis by weakening the survival mechanism of HSC-3 through the regulation of these two genes. In comparison, the chemotherapeutic drug cisplatin significantly enhanced the expression of *Bax* and suppressed the expression of *Bcl-2* in a greater manner than GUHE, indicating a more prominent effect than *G. umbrosus* . The greater expression of both *Bax* and *Bcl-2* by cisplatin was predictable, given its conventional role as a potent chemotherapeutic drug for oral cancer. 24 However, findings demonstrated that GUHE produced comparable effects, indicating a similar potential to cisplatin in inducing apoptosis in HSC-3 cells.

The ratio between *Bax* and *Bcl-2* is vital in initiating the mitochondrial apoptosis pathway, where *Bax* triggers apoptosis while *Bcl-2* opposes this mechanism. 25 The induction of the mitochondrial apoptosis pathway by a potential anticancer agent was closely related to the inhibition of *Bcl-2* function, which suggests that the inhibition of this gene might be detrimental to cancer cells. 23

Current findings of upregulated *Bax* and downregulated *Bcl-2* in HSC-3 cells indicate that GUHE induced apoptosis, as it eradicates the malignant cells by restoring the programmed cell death, which is often dysregulated in cancer cells. These findings were consistent with a previous report where *G. lanceolatus* upregulated the expression of *Bax* gene and downregulated *Bcl-2* expression in the MCF-7 cell line. 26 In addition, *G. macrophyllus* was also reported to significantly enhance the expression of the *Bax* gene in Swiss albino mice inoculated with Ehrlich ascites carcinoma (EAC). 27

Interestingly, the HGF cells treated with GUHE did not significantly affect the regulation of *Bax* and *Bcl-2,* reflecting that GUHE did not induce apoptosis in HGF cells and exert selective cytotoxicity toward HSC-3 without harming the HGF cells. This selectivity is a preferable approach in the development of anti-cancer agents to minimize the damage to normal healthy tissue, making it a promising candidate as adjuvant therapy for oral cancer, particularly for patients with side effects from conventional chemotherapy.

The regulation of *Bax* and *Bcl-2* genes supports the activation of the mitochondrial apoptotic pathway by GUHE, where the upregulation of *Bax* will increase the membrane permeability, leading to subsequent activation of caspase-3 and eventually triggering apoptosis. 19

Caspase-3 plays a crucial role in the execution phase of the mitochondrial apoptosis pathway. 28 The activation of caspase-3 further cleaves the important cellular components, leading to cell death. The modulation of the *Bax* / *Bcl-2* ratio, coupled with the activation of caspase-3, appeared to be the key strategy in inducing apoptosis in cancer cells.

The caspase-3 activity assay was quantified as a key regulator of apoptosis induction by GUHE in the HSC-3 cell line. Caspase-3 is an important executioner that triggers the apoptosis pathway by cleaving its protein substrates. It was discovered that the concentration of caspase-3 was significantly increased in HSC-3 treated with GUHE and cisplatin compared to the control, indicating GUHE triggered a cell death cascade through an apoptosis pathway similar to cisplatin. An increase in the concentration of caspase-3 protein in the HSC-3 cells treated with GUHE and cisplatin depicts an increase in the apoptotic activity in this cell line. These results were consistent with earlier results of upregulation of *Bax* and downregulation of *Bcl-2* in GUHE-treated HSC-3.

The activation of caspase-3 as an apoptotic executioner in this mitochondrial apoptosis pathway plays a vital role against cancer cells. 22 The activated caspase-3 triggers apoptosis by cleaving the substrate poly (ADP-ribose) polymerase (PARP) and lamin, leading to the alteration of cellular morphology and biochemistry. 22 The stimulation of this caspase cascade via the mitochondrial pathway has also been proven in previous studies as a mechanism for targeted alternative therapy induced by traditional medicine. 29 30 Captivatingly, the concentration of caspase-3 in HGF treated with GUHE was not significantly regulated compared to the control.

The findings from the current study proved that GUHE induced the mitochondrial apoptotic pathway in the HSC-3 cell line via upregulation of *Bax* , downregulation of *Bcl-2* , and activation of caspase-3. It also revealed that the cytotoxic effect and apoptosis induction of GUHE were selective in targeting only HSC-3 cells while exerting an insignificant effect on HGF cells.

Targeting apoptosis in OSCC is vital in developing effective therapeutic strategies that minimize the damage to normal cells while maximizing the eradication of cancer cells. This approach aims to exploit the biological features of OSCC, allowing for the apoptosis induction in malignant cells without harming the surrounding healthy cells. Understanding the underlying mechanism of targeted cytotoxicity is crucial as it leads to innovative therapy that can enhance patient outcomes, reduce the side effects, and improve the overall quality of life for oral cancer patients.

The limitation of the current study was that the experiments were conducted *in vitro* , which may not fully capture the complexity of the tumor microenvironment. *In vitro* models are valuable for elucidating the cellular responses; however, they lack critical interactions with surrounding tissues, vasculature, and immune components that play significant roles in tumor progression and therapeutic response. To better comprehend the therapeutic potential of *G. umbrosus* , future studies that employ *in vivo* models are highly recommended, as this model will provide a more accurate and comprehensive evaluation of efficacy and safety.

## Conclusion

These cytotoxic effects of GUHE are mediated through the induction of the mitochondrial apoptosis pathway, as proved by the upregulation of *Bax* , downregulation of *Bcl-2* , and activation of caspase-3 protein in the HSC-3 cell line after exposure to GUHE, with insignificant impact on the HGF cell line. Collectively, current findings highlight the potential anticancer effect of *G. umbrosus* in oral cancer. Further research needs to be conducted to identify the possible bioactive compound responsible for the observed effects and explore its potential as a targeted adjuvant anticancer drug.

## References

[1] Ferlay J Ervik M Lam F Global Cancer Observatory: Cancer Today (version 1.1)Lyon, France: International Agency for Research on Cancer. Published 2024. Accessed August 23, 2024 at:https://gco.iarc.who.int/today

[2] Zhang S Z Xie L Shang Z J Burden of oral cancer on the 10 most populous countries from 1990 to 2019: estimates from the Global Burden of Disease Study 2019 Int J Environ Res Public Health 2022 19 02 875 35055693 10.3390/ijerph19020875 PMC8775770

[3] Tan Y Wang Z Xu M Oral squamous cell carcinomas: state of the field and emerging directions Int J Oral Sci 2023 15 01 44 37736748 10.1038/s41368-023-00249-w PMC10517027

[4] Jeihooni A K Jafari F Oral cancer: epidemiology, prevention, early detection, and treatment IntechOpen 2021 10.5772/intechopen.99236

[5] Chang H P Huang M C Lei Y P Chuang Y J Wang C W Sheen L Y Phytochemical-rich vegetable and fruit juice alleviates oral mucositis during concurrent chemoradiotherapy in patients with locally advanced head and neck cancer J Tradit Complement Med 2022 12 05 488 498 36081822 10.1016/j.jtcme.2022.03.004 PMC9446194

[6] Wani A K Akhtar N Mir T UG Targeting apoptotic pathway of cancer cells with phytochemicals and plant-based nanomaterials Biomolecules 2023 13 02 1 34 10.3390/biom13020194 PMC9953589 36830564

[7] Senga S S Grose R P Hallmarks of cancer - The new testament Open Biol 2021 11 01 200358 33465324 10.1098/rsob.200358 PMC7881179

[8] Mali S B Cancer treatment: role of natural products. Time to have a serious rethink Oral Oncol Rep 2023 6(April):100040

[9] Kamaruddin Mat-Salleh & AL Tumbuhan Ubatan Malaysia Bangi Pusat Pengurusan Penyelidikan UKM 2002

[10] Toha Z M Zainoddin H N Wan Aziz W N Arsad H Antiproliferative effect of the methanol extract of *Goniothalamus umbrosus* leaves on Hela cells J Biol Sci Opin 2018 6 02 17 22 10.7897/2321-6328.06276

[11] Wan Ghazali WA Ab Alim A Kannan T P Ali N AM Abdullah N A Mokhtar K I Anticancer properties of Malaysian herbs: a review Arch Orofac Sci 2016 11 02 19 25

[12] Wiart C Goniothalamus species: a source of drugs for the treatment of cancers and bacterial infections? Evid Based Complement Alternat Med 2007 4 03 299 311 17965760 10.1093/ecam/nem009 PMC1978243

[13] Che Aziz N Alahmad B EM Kashmoola M A Lestari W Mokhtar K I Nik Mohd Rosdy N MM *Goniothalamus umbrosus* induces cell cycle arrest in oral squamous cell carcinoma cell line J Int Dent Med Res 2024 17 03 996 999

[14] Poonacha S K Harishkumar M Radha M Insight into oroxylina-7-o-β-d-glucuronide-enriched oroxylum indicum bark extract in oral cancer hsc-3 cell apoptotic mechanism: role of mitochondrial microenvironment Molecules 2021 26 24 1 19 10.3390/molecules26247430 PMC8704017 34946511

[15] Abdelwahab S I Abdul A B Elhassan M M Biological and phytochemical investigations of *Goniothalamus umbrosus* leaves hexane extract J Med Plants Res 2009 3 11 880 885

[16] Agatonovic-Kustrin S Morton D W Mizaton H H Zakaria H The relationship between major polyphenolic acids and stigmasterol to antioxidant activity in different extracts of *Myrmecodia platytyrea* S Afr J Bot 2018 115 94 99

[17] Aziz N C Alahmad B EM Kashmoola M A Lestari W Rosdy N MMNM Mokhtar K I Oral cancer's new enemy: *Goniothalamus umbrosus* targets oral squamous cell carcinoma and spare human gingival fibroblast cells Eur J Dent 2025 19 02 457 463 39788531 10.1055/s-0044-1801278 PMC12020607

[18] Sordi M B Panahipour L Gruber R Oral squamous carcinoma cell lysates provoke exacerbated inflammatory response in gingival fibroblasts Clin Oral Investig 2023 27 08 4785 4794 10.1007/s00784-023-05107-x PMC10415472 37391526

[19] He S Chakraborty R Ranganathan S Proliferation and apoptosis pathways and factors in oral squamous cell carcinoma Int J Mol Sci 2022 23 03 1562 35163485 10.3390/ijms23031562 PMC8836072

[20] Roshanravan N Asgharian P Dariushnejad H *Eryngium billardieri* induces apoptosis via *Bax* gene expression in pancreatic cancer cells Adv Pharm Bull 2018 8 04 667 674 30607339 10.15171/apb.2018.075 PMC6311640

[21] Spitz A Z Gavathiotis E Physiological and pharmacological modulation of BAX Trends Pharmacol Sci 2022 43 03 206 220 34848097 10.1016/j.tips.2021.11.001 PMC8840970

[22] Nazeri M Mirzaie-Asl A Saidijam M Moradi M Methanolic extract of Artemisia absinthium prompts apoptosis, enhancing expression of Bax/Bcl-2 ratio, cell cycle arrest, caspase-3 activation and mitochondrial membrane potential destruction in human colorectal cancer HCT-116 cells Mol Biol Rep 2020 47 11 8831 8840 33141288 10.1007/s11033-020-05933-2

[23] Singh R Letai A Sarosiek K Regulation of apoptosis in health and disease: the balancing act of BCL-2 family proteins Nat Rev Mol Cell Biol 2019 20 03 175 193 30655609 10.1038/s41580-018-0089-8 PMC7325303

[24] Bijani F Zabihi E Bijani A Nouri H R Nafarzadeh S Seyedmajidi M Evaluation of apoptotic effect of crocin, cisplatin, and their combination in human oral squamous cell carcinoma cell line HN5 Dent Res J (Isfahan) 2021 18 70 34584648 PMC8428283

[25] Pistritto G Trisciuoglio D Ceci C Garufi A D'Orazi G Apoptosis as anticancer mechanism: function and dysfunction of its modulators and targeted therapeutic strategies Aging (Albany NY) 2016 8 04 603 619 27019364 10.18632/aging.100934 PMC4925817

[26] Nasibah R The potential anticancer effects of Goniothalamus lanceolatus extracts in inducing apoptosis in breast cancer (MCF-7) and ovarian cancer (PEO1 AND PEO4) cell linesMasters Thesis. Universiti Teknologi MARA (UiTM); 2021. Accessed October 21, 2025 at:https://ir.uitm.edu.my/id/eprint/60456

[27] Abd El-Kaream S A Hussein N GA El-Kholey S M Elhelbawy A MAEI Microneedle combined with iontophoresis and electroporation for assisted transdermal delivery of *Goniothalamus macrophyllus* for enhancement sonophotodynamic activated cancer therapy Sci Rep 2024 14 01 7962 38575628 10.1038/s41598-024-58033-7 PMC10994924

[28] Eskandari E Eaves C J Paradoxical roles of caspase-3 in regulating cell survival, proliferation, and tumorigenesis J Cell Biol 2022 221 06 e202201159 35551578 10.1083/jcb.202201159 PMC9106709

[29] Hsiao Y C Sheng Y H Chen T Y Leu W J Hsu J L Hsu L C Lin L C Guh J H Repurposing Suan-Zao-Ren-Tang as anticancer agents in apoptotic sensitization against non-small cell lung cancer cell lines through amplification of spindle assembly checkpoint activation J Tradit Complement Med 2024 15 06 601 614 41169944 10.1016/j.jtcme.2024.08.008 PMC12570107

[30] Lai C I Chu Y L Ho C T Su Y C Kuo Y H Sheen L Y Antcin K, an active triterpenoid from the fruiting bodies of basswood cultivated *Antrodia cinnamomea* , induces mitochondria and endoplasmic reticulum stress-mediated apoptosis in human hepatoma cells J Tradit Complement Med 2015 6 01 48 56 26870680 10.1016/j.jtcme.2014.11.026 PMC4737972

